# Large-Area Two-Dimensional Plasmonic Meta-Glasses and Meta-Crystals: a Comparative Study

**DOI:** 10.1007/s11468-016-0397-9

**Published:** 2016-10-08

**Authors:** Stefano De Zuani, Marcus Rommel, Ralf Vogelgesang, Jürgen Weis, Bruno Gompf, Martin Dressel, Audrey Berrier

**Affiliations:** 1 Physikalisches Institut and Research Center SCoPE, Universität Stuttgart, Pfaffenwaldring 57, 70569 Stuttgart, Germany; 2 Max Planck Institute for Solid State Research, Heisenbergstrasse 1, 70569 Stuttgart, Germany; 3 Present Address: Chalmers University of Technology, MC2 Kemivägen 9, 41285 Gothenburg, Sweden; 4 Institut für Physik, Carl von Ossietzky Universität Oldenburg, Ammerländer Heerstraße 114-118, 26129 Oldenburg, Germany

**Keywords:** Plasmonics, Localized surface plasmons, Random/ordered arrays, Coupled dipole approximation, Metallic nanoparticle arrays

## Abstract

The geometrical arrangement of metallic nanoparticles plays a crucial role on the optical response of nanoplasmonic samples due to particle-particle interactions. In this work, large-area, two-dimensional meta-glasses (random arrangements) and meta-crystals (periodic arrangements) made of identical metallic nanoparticles are investigated for three different particle densities of 5, 10, and 15 discs/μm^2^. A direct comparison between random and periodically ordered arrays is presented. The comparison clearly shows that the particle density has the largest influence on the extinction spectra for both periodic and random samples, and that for equal densities, the optical response away from diffraction effects is strikingly similar in both cases. The role of the radial density function and minimum particle distance is also determined. This study elucidates the role of the particle-particle interactions on the response of plasmonic nanoparticles and indicates how to control position and shape of the plasmonic resonance.

## Introduction

Localized surface plasmon polaritons (LSPPs), generated by light coupled to the conductive electrons of metallic nanoparticles, are of great interest for many applications in the context of future optics and electronics [1–4]. The excitation of LSPP of a single isolated metallic nanoparticle is characterized by resonance peaks in the extinction spectra referred to as localized surface plasmon resonances (LSPRs) and leads to a strong, confined field enhancement in the surrounding of the particle. It is well known that the shape and the position of the LSPR peak can be modified by varying the size [5], shape [6], and material properties [7] of the nanoparticle as well as of the surrounding medium [8]. Measuring the far-field optical response of a single particle is not easy in practice since the spot size of the incident light beam is usually much larger than the extinction cross section of the single particle leading to very weak signals. When metallic nanoparticles are closely placed with respect to each other, their interaction induces changes in the local field, which modifies the overall resonance. The simplest illustration is that of a dimer antenna where two nanoparticles are placed in vicinity and separated by a small gap [9]. When large particle arrays are required, periodic lattices are commonly used. However, periodic arrangements introduce special features originating from the regular arrangement, mainly due to the presence of Rayleigh-Woods anomalies (RWA) [10]. Therefore, a common solution is to increase the particle density by placing the individual nanoparticles in a random arrangement in order to average the particle-to-particle interactions out, and hence boost the single particle resonance signal. It is believed that the resulting resonance reflects the shape and position of the single particle resonance, provided the array is large enough [11]. Hitherto, little is known about the respective influence of short-range and long-range particle-particle interactions on the plasmonic resonance of a large two-dimensional field of randomly arranged nanoparticles placed on a dielectric substrate. Recently, it was theoretically shown that the center-to-center particle distance influences the position and amplitude of the extinction of an amorphous array of metallic nanoparticles, [12] and it was discussed that the long-range dipole-dipole interaction in disordered patterns is still present when the center-to-center particle distance reaches a few times the disc diameter [13]. However, a unified view on the dipole-dipole interactions in both ordered and random patterns is still lacking.

When nanoparticles are placed in a periodic, two-dimensional (2D) lattice, the particle-particle interactions induce a reshaping of the LSPR when collective modes are excited around the positions of the RWA. The coherently accumulated phase between neighboring particles plays a dominant role leading to modifications of the resonance peak compared to the single particle response. By acting on the inter-particle distance (i.e., the period) in the array, the dipolar interaction between particles can be consequently changed resulting in strong modifications of the bandwidth and position of the resonance peak for different grating constants [14]. When the inter-particle distance is comparable to the particle size, dipole-dipole interactions between individual particles lead to spectral shifts in the extinction of ensembles of particles, which sensitively depend on the distance [15]. Trying to eliminate the effect of coherent radiative interactions by increasing the particle separation even more will introduce diffraction modes which strongly modify the extinction resonance of the array [16]. If the particle distance is chosen in such a way that a RWA of the array falls in proximity of the LSPR of the individual particle, extremely narrow resonances can be achieved [17, 18]. Although the RWA feature is very well documented, less is known on the particle-particle interactions away from these anomalies.

Introducing disorder has been an attempt to reduce the influence of the periodicity and come closer to the behavior of the LSPR of the single nanoparticle. In this case, randomness ensures the lack of long-range order among the particles and the absence of coherent grating interference. Broader and weaker extinction spectra compared to ordered arrays have been reported [19]. There is a continuous effort in exploring the optical response of metallic nanoparticles in amorphous arrays [19–21]. In particular, interesting is the question of the influence of the collective interaction of the long distance particles compared to that of the nearest neighbors. The position and linewidth of the extinction spectra of amorphous arrays of nanodiscs depend on the minimum allowed particle-particle separation, and oscillations in the resonance position were found [22]. Some works have also investigated the influence of the nearest neighbor distance in random samples [12, 13, 23] and found a variation on the position of the resonance as a function of the smaller inter-particle distance [22]. However, most of the studies were limited to normal incidence investigations while a clear wavevector (***k***) dependence of the optical response is expected for interacting nanostructures [24]. Some questions are therefore raised: what is the influence of the particle density and long-range interaction, while keeping the nearest neighbor distance constant? How does the particle-particle distance distribution affect the resonances of periodic compared to random arrangements in terms of position, shape, and dispersion of the LSPR?

In this article, we compare the optical response of periodic arrangements, or “meta-crystals,” to similar density random arrangements, or “meta-glasses.” We identify the role of the density distribution of the nanoparticles of both meta-crystals and meta-glasses on their optical response in a broad spectral range and large **k**-space by experimentally comparing samples measured with light beams with highly defined **k**-vectors to coupled dipole approximation calculations. We find that the optical response of these particle arrangements is more influenced by the particle density than by the nature of the arrangement itself, provided that we stay away from the RWA. The main parameters are the local particle density and the nearest neighbor distance. To the best of our knowledge, this is the first work where a rigorous comparison of the optical response of ordered and random arrays of identical gold nanodiscs with controlled minimum distance and well-defined **k** of the incident beam is reported. This study sheds new light on the environment-related fluctuations of the plasmonic resonance of nanoparticles and is relevant to applications such as light enhancement, photovoltaics, and sensing applications.

## Random Vs Periodic Arrangements: Experimental

**Fig. 1:**
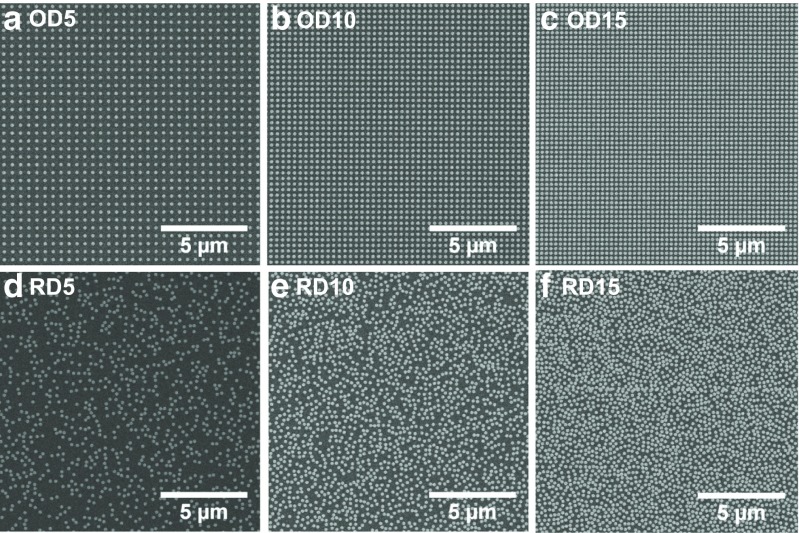
Scanning electron microscope images of a 15 × 15 μm^2^ zoomed areas of the six fabricated gold nanodisc arrays. The ordered nanodisc arrays (*top row*) and the random arrays (*bottom row*) are shown with density of 5 discs/μm^2^, OD5 in **a** and RD5 in **d**; 10 discs/μm^2^, OD10 in **b** and RD10 in **e**; and 15 discs/μm^2^, OD15 in **c** and RD15 in **f**

**Fig. 2:**
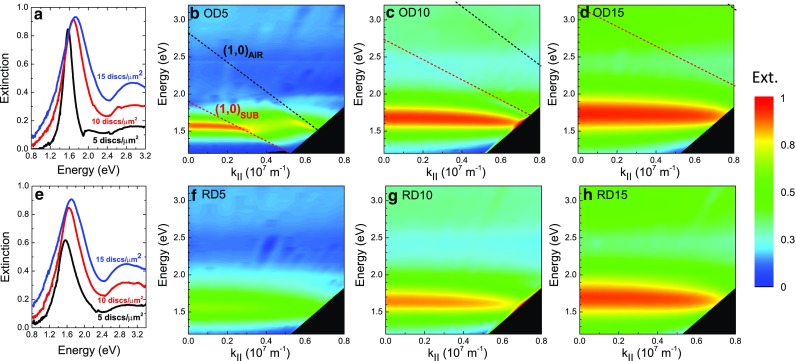
Experimental contour plots of the extinction spectra obtained from the transmittance measurements of the fabricated gold nanodisc arrays with p-polarized light at different parallel wavevector components. **a** and **e** represent the extinction line cuts at normal incidence as a function of the particle density for, respectively, the ordered and random cases. In the *top row*, the ordered nanodisc arrays are shown with density of **b** 5 discs/μm^2^, **c** 10 discs/μm^2^, and **d** 15 discs/μm^2^. In the *bottom row*, the random nanodisc arrays are shown with density **f** 5 discs/μm^2^, **g** 10 discs/μm^2^, and **h** 15 discs/μm^2^. Rayleigh-Wood’s diffraction modes (1,0)_AIR_ and (1,0)_SUB_ are represented in **b**, **c**, and **d**, respectively, in *black and red dashed lines*

The investigated samples consist of three pairs of either ordered or random nanodisc arrays of density 5, 10, and 15 discs/μm^2^, respectively, fabricated on a fused silica substrate by means of electron beam lithography. Rigorous sample fabrication of the arrays is crucial in order to ensure a very narrow particle size distribution and avoid unwanted red-shift and broadening of the extinction resonance. The precise control of our processing steps yields well-defined nanodisc diameters independent on the particle density and insures the absence of connected dimers. The large-area fields of 1.5 × 1.5 mm^2^ allow us to fully characterize the optical response of our samples, by performing measurements over a broad range of angles of incidence and frequencies by means of a free-space, variable-angle spectroscopic transmission setup with collimated light beam (see “Experimental Section”). Scanning electron microscope images of a sample area of 15 × 15 μm^2^ for the six different samples are shown in Fig. 1. One has to underline that only the arrangement is modified between both parts of a pair a/d, b/e, and c/f; the particle size as well as the density is kept the same. The gold nanodiscs have a height of 30 nm and a diameter of 190 nm, and, in the case of the ordered arrays, they are arranged in a square periodic array of lattice constant 262, 312, and 439 nm for the OD5, OD10, and OD15, respectively, as measured from the SEM images. For the randomly distributed nanodiscs, special care was taken to avoid the presence of merged particles by enforcing a minimum center-to-center distance of 210 nm. Whereas in Fig. 1d, e, no touching particles are observed; Fig. 1f shows the presence of few aggregates along the stitching lines between two different e-beam writing fields. The amount of stitching-related dimers is negligible when the whole sample area is considered and it does not affect the final optical response of the sample. This is confirmed by the absence of any dimer-related features at energies lower than the single dipolar resonance in the measured spectra. The samples were investigated with intensity transmittance measurements using p-polarized incident light between 350 nm (3.54 eV) and 1500 nm (0.826 eV), varying the angle of incidence from 0 to 60°. The dependence of the extinction *Ext*, where *Ext* = 1–*T*, with *T* the measured transmittance, on the wavevector **k** of the p-polarized incident radiation is shown for the six nanodisc arrays in the contour plots of Fig. 2 in the energy range between 0.826 and 3.2 eV. All spectra are dominated by a strong LSPR around 1.65 eV due to the main dipolar excitation of the nanodisc ensemble. In Fig. 2, a similar trend is seen for both meta-crystals (Fig. 2b–d) and meta-glasses (Fig. 2f–h): the intensity of the extinction peak increases with increasing density and it becomes broader. Major deviations from this trend are seen in the ordered nanodisc arrays, for which the modifications of the LSPR are induced by the RWA, especially at the lowest density (Fig. 2b). The dipolar coupling between the evanescent diffraction mode and the LSPRs of the individual particles leads to the development of a new and very narrow plasmonic resonance well known as surface lattice resonance (SLR). [11, 25–27] Both RWAs in the substrate and in the air semi-space are visible in Fig. 2b: a clear weakening of the intensity with increasing wavevector and a distortion of the normal Lorentzian shape of the LSPR is shown due to the effect of the (1,0) air mode and in particular of the (1,0) substrate mode. The semi-embedding of the nanoparticles translates in an average refractive index of 1.25 for the single particle resonance spectral position; however, the position of the RWA is decided by the real refractive indices, *n*_*sub*_ = 1.49 for the glass substrate and *n* = 1 for air according to the equation: $E=\frac{\hslash c}{n}\left|{k}_{\parallel }+m{G}_x+w{G}_y\right|$, where *m* and *w* are integers, *G*_*x,y*_ are reciprocal space vectors, *k*_∥_ is the projection of the wavevector onto the sample surface, *n* is the refractive index of the environment, *c* is the speed of light in vacuum, and *ℏ* is Planck’s constant [28]. With increasing density of the arrays, the diffraction modes shift to higher energies further away from the LSPR. In Fig. 2c, the (1,0) substrate mode is still coupled to the LSPR at large **k**-vectors; instead for the ordered array OD15 of Fig. 2d, the diffraction modes are too far away in energy and their effect on the LSPR is negligible, allowing an easier comparison between the optical response of random and ordered array. A very similar dispersion of the extinction resonance is seen for both random and ordered arrays in the case of 10 discs/μm^2^, and especially 15 discs/μm^2^. The optical response of the plasmonic arrays seems independent of the specific arrangement of the nanodiscs and is more influenced by the particle density.

## Coupled Dipole Approximation Applied to Meta-Crystals and Meta-Glasses

Coupled dipole approximation (CDA) is a well-known and effective method to calculate the extinction cross section of plasmonic particles as a function of distance to their neighbors. [29] In the CDA framework, each nanodisc is modeled by a point dipole excited by an external electromagnetic field. The coupling between the radiating field of the point dipole and the other dipoles present in its surroundings is also taken into account. The simplest case is the case of a single isolated particle: its static polarizability is expressed as

1 $$ {\alpha}_0=\frac{\left({\varepsilon}_p-{\varepsilon}_b\right)}{\left[L\left({\varepsilon}_p-{\varepsilon}_b\right)+{\varepsilon}_b\right]}V\kern0.5em , $$

where *ε*_*p*_ and *ε*_*b*_ are the permittivities of the particle and of the background, respectively, *L* is the depolarization factor taking into account the shape of the ellipsoidal particle with respect to the field orientation, and *V* is the volume of the particle. For larger particles, effects of depolarization and radiative damping effect are taken into account through the modified long wavelength approximation [30] with *k* the wavevector of the incident light, *R* the radius of the nanoparticle in the direction of polarization, and

2 $$ \alpha =\frac{\alpha_0}{1-{\alpha}_0\frac{k^2}{R}-\frac{2}{3}i{k}^3{\alpha}_0}. $$

When many particles are placed in an array, dipolar interactions in between particles placed at a distance *r*_*ij*_ from each other need to be taken into account through the structure factor *S*. In the most common case, the arrangement is periodic and the results are well known [31] as follows:

3 $$ {S}_{per}={\displaystyle \sum_{j\ne i}}{e}^{ik{r}_{ij}}\left[\frac{\left(1-ik{r}_{ij}\right)\left(3{ \cos}^2{\theta}_{ij}-1\right)}{r_{ij}^3}+\frac{k^2{ \sin}^2{\theta}_{ij}}{r_{ij}}\right]. $$

with *r*_*ij*_ the distance between particles *i* and *j*, and *θ*_*ij*_ the angle between *r*_*ij*_ and the polarization direction.

Antosiewicz et al. have recently shown [22] that a similar formulation is valid in the case of random arrangements and expressed the structure factor *S* as

${S}_{ran}={\displaystyle \underset{C}{\overset{+\infty }{\int }}}{\displaystyle \underset{0}{\overset{2\pi }{\int }}}{e}^{ikr}\left[\frac{\left(1-ikr\right)\left(3co{s}^2\theta -1\right)}{r^3}+\frac{k^2si{n}^2\theta }{r}\right]G\left(r,C,\upsigma \right) rd\theta dr,$ which after integration over the whole two-dimensional (*r*,*θ*) space, is expressed as

4 $$ {S}_{ran}=\pi \sigma {\displaystyle \underset{C}{\overset{+\infty }{\int }}}{e}^{ikr}\left({k}^2-\frac{1-ikr}{r^2}\right)rdf\left(r,\sigma, C\right)dr, $$

where *G*(*r*, *C*, σ) = *σ rdf*(*r*, *σ*, *C*) and the radial density function *rdf*(*r*, *σ*, *C*) is a function describing the evolution of the number of neighboring particles as a function of the radial distance *r* from the considered particle. *C* is the minimum nearest neighbor distance (center-to-center) and *σ* the particle density. This function is strongly dependent on the sample fabrication routine and will be described in the next section. Equation (4) is valid at normal incidence. In order to take dispersion into account, we modify the phase factor into *e* ^(i *k r* (1+sin(*α*))),^ with *α* the angle of incidence [32]. This phase factor takes now into account the apparent distance between particles when the angle of incidence increases. Introducing this apparent distance is an effective parameter to reproduce the measured dispersion. It is already interesting to note that the expressions for *S*_*per*_ and *S*_*ran*_ are very similar, as the dipole-dipole interactions are in both cases described by the Green dyadic. This gives us a hint that the influence of the surrounding particles could be similar. Once the polarizability function is known, the extinction efficiency can be expressed in the single particle case as

5 $$ {Q}_{ext\_ \sin gle}=\frac{4\pi k}{\pi {R}^2}Im\left(\alpha \right) $$

and, in the interacting case, as

6 $$ {Q}_{ext}=\frac{4\pi k}{\pi {R}^2}Im\left(\frac{1}{\alpha^{-1}-S}\right). $$

At oblique incidence, a dependency of cos*θ* with *θ* being the angle of incidence is introduced in Eq. (6), in order to take into account the orientation of the polarization of the exciting field in respect to the plane of incidence when p polarization is used.

The radial density function (RDF) *rdf*(*r*, *σ*, *C*) defines the number of neighboring nanoparticles of radius *R*_*disc*_ integrated over 2π at a certain distance from a central particle taken as origin.

**Fig. 3:**
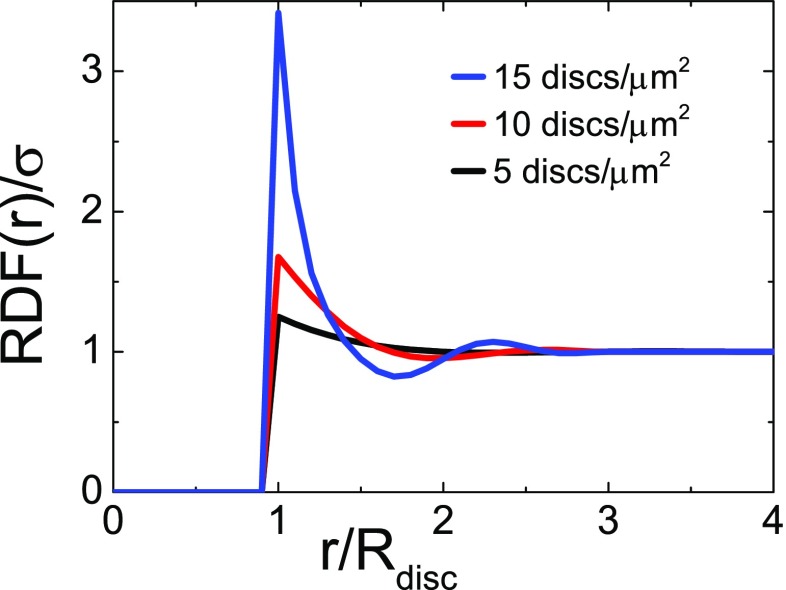
Radial density function RDF_exp_ normalized to the particle density σ for the three samples fabricated in this study, as a function of the center-to-center distance between particles *r* normalized to the disc radius *R*_*disc*_

Figure 3 displays the experimental RDF as obtained from the generated random patterns used to design our samples. The RDF is strongly varying up to three times the particle radius. The strongest fluctuations are seen for the denser samples. At larger distances, the RDF is equal to the average particle density σ. The samples fabricated in this work follow a certain RDF; to distinguish it from other RDFs used later in the simulations, we call the experimentally realized one RDF_*exp*_. It is zero up to a normalized nearest neighbor distance of *r/R*_*disc*_ = 1 and then shoots up to values well above 1, depending on the density, i.e., in the direct vicinity of each particle there are more neighbors than expected from the averaged value.

## Comparison Experimental Calculations

**Fig. 4:**
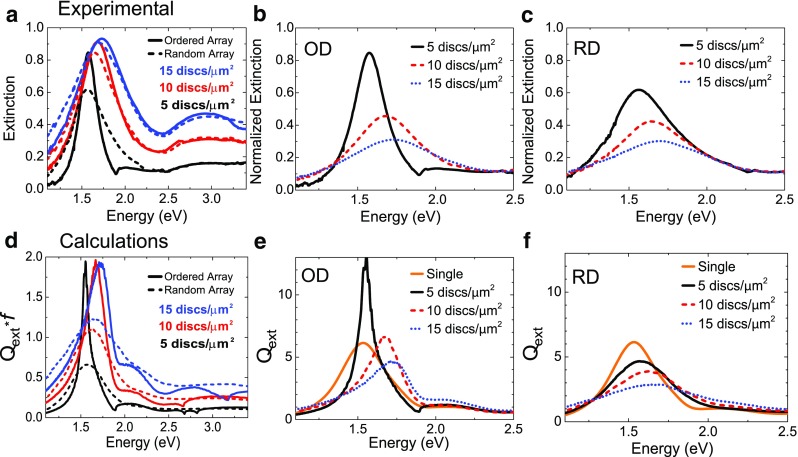
Comparison of measured and calculated extinction of p-polarized incident light at normal incidence for periodic vs random samples. **a** Measured extinction for random (*dashed line*) and ordered (*solid line*) nanodisc arrays. Measured extinction normalized to a density of five discs/μm^2^ for ordered nanodisc arrays (**b**) and random nanodiscs arrays (**c**). **d** Calculated extinction efficiency *Q*_*ext*_ multiplied by the filling factor *f* for random (*dashed line*) and ordered (*solid line*) nanodisc arrays. **e** Calculated extinction efficiency *Q*_*ext*_ for ordered (OD) nanodisc arrays. **f** Calculated extinction efficiency *Q*_*ext*_ for random (RD) nanodisc arrays. In all figures, samples with different densities are represented with *black* (five discs/μm^2^), *red* (10 discs/μm^2^), and, respective, *blue* (15 discs/μm^2^) *lines*. The case of non-interacting particles (“Single”) is also added with an *orange line*

The comparison between the measured and the calculated optical properties for **k** = 0 (normal incidence) is shown in Fig. 4. Figure 4a shows the total extinction of each sample, as measured. It is striking to note the similarities, at constant particle density, between the resonances of the meta-glasses and the meta-crystals both in amplitude and resonance position. This gives us a first hint that the resonance shift is probably dominated essentially by the background particle density and not so much by the specific arrangement of the particles themselves. Comparing the results at normal incidence for both the random and ordered arrays as shown in Fig. 4a, we can see that the LSPR occurs for the periodic arrays at 1.57 eV (OD5) vs at 1.56 eV (RD5), 1.68 eV (OD10) vs 1.64 eV (RD10), and 1.73 eV (OD15) vs 1.70 eV (RD15). The width of the extinction peak is systematically larger in the random case especially in the case where the RWA modifies the resonance shift. In order to compare the influence of the particle environment on a given particle, we divide the experimental spectra by their respective filling factors *f*, as shown in Fig. 4b, c. The used effective filling factors are given in Table 1. In Fig. 4b, c, the normalized extinction (extinction per particle) is plotted for the periodic and random samples separately. In both cases, the extinction per particle strongly decreases when the particle density increases. The large extinction experienced for density five discs/μm^2^ is due to the presence of the diffraction modes.

**Table 1:** Effective filling factors of the fabricated nanostructures for the three densities and ordering

Density	5/μm^2^ (%)	10/μm^2^ (%)	10/μm^2^ (%)
Ordered	*f* = 14.7	*f* = 29.1	*f* = 41.3
Random	*f* = 14.2	*f* = 28.4	*f* = 42.5

For comparison, the results of the calculations are shown in Fig. 4d–f. In order to compare the relative extinction efficiencies with the measurements, in Fig. 4d, we plot the normalized extinction efficiency where the calculated *Q*_*ext*_ is multiplied by the filling fraction *f*. In this way, the differences of extinction due to different densities of particles are taken into account. The position and the shape of the resonance peaks for both ordered and random nanodisc arrays and the overall behavior of the resonance with the increasing particle density resulting from the calculation agree reasonably well with the results obtained from the measurements. The reported data in Fig. 4b, c and Fig. 4e, f provide us with important information regarding the role of the interaction between neighboring nanodiscs on the final response of the nanodisc arrays. The single particle resonance, which represents the non-interacting case, is added for comparison in Fig. 4e, f. The extinction per particle decreases when the particles are more densely packed. This gradual decrease with particle density is also shown in the calculated spectra (Fig. 4e, f). The ordering seems here to have a smaller effect than the density as a similar trend is seen both for the meta-crystals and the meta-glasses. The broadening and blue shift of the resonance peak position is clearly visible for both ordered and random arrays; it is attributed to the increase of the collective radiative dipole coupling interaction among adjacent nanodiscs with decreased particle distance. A similar shift is obtained for meta-glasses and meta-crystals, with the main influencing parameter being the particle density. When looking more closely, two main influences can be isolated as follows: (a) the influence of the nearest neighbors and (b) that of the background density of all other particles. In Fig. 4f, the single resonance of a nanodisc in the array RD5 peaks almost at the same wavelength as that for a completely isolated nanodisc. In RD15 instead, a clear broadening and shift of the extinction peak occurs. Interestingly, this behavior is mainly dependent on the overall particle density and less on the exact position of the particles. It is worth reminding at this point that the minimum inter-particle distance is constant for all the random arranged nanodiscs, and, hence, possible differences in the shape and position of the extinction peak deriving from changing in the minimum allowed particle distance between the samples can be excluded [22].

**Fig. 5:**
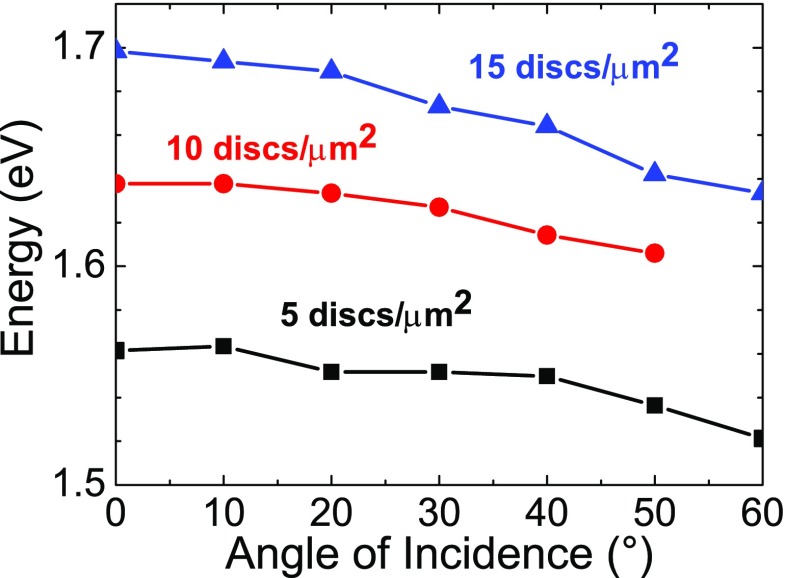
Measured position of the LSPR of the random nanodisc arrays with increasing angle of incidence for p-polarized light. Samples with different densities are indicated using *black* (RD5), *red* (RD10), and *blue* (RD15) *lines*

A second important point to be discussed is the role of dispersion in the particle arrays. Three-dimensional, isolated, and non-interacting nanoparticles supporting LSPRs are not affected by dispersion; irrespective of the incident wavevector, they resonate at the same frequency. This is not the case anymore when the nanoparticles are interacting; they experience a **k**-dependence which can be measured by the energy shift of the peak maximum of the extinction as a function of the angle of incidence of the light onto the sample. As can be seen already from Fig. 2, the dispersion is very similar for the ordered and random samples, except for the RWA appearing in the ordered samples of lower density. The measured shift of the LSPR for the meta-glass samples of different densities is displayed on Fig. 5. The resonance at normal incidence (**k** = 0) shifts to the blue when the density increases, as already discussed above, and the dispersion increases with density. A clear red shift of the resonance position linearly increasing with the angle of incidence is visible in Fig. 5 for the random arrays. The shift of the resonance to lower energies with increasing angle of incidence is more pronounced for the RD15 than that for the RD5. At high density, the proximity of a larger number of neighboring particles increases the retardation effects and the scattered electric fields generated by the dipoles reduces the restoring force on the electrons in the neighboring particle, thereby red shifting the resonance frequency. At higher angles of incidence, the total number of nanodiscs contributing to the final optical response is larger than that at low angles of incidence, hence increasing the red shift.

## Influence of the Radial Density Function and Nearest Neighbor Distance

**Fig. 6:**
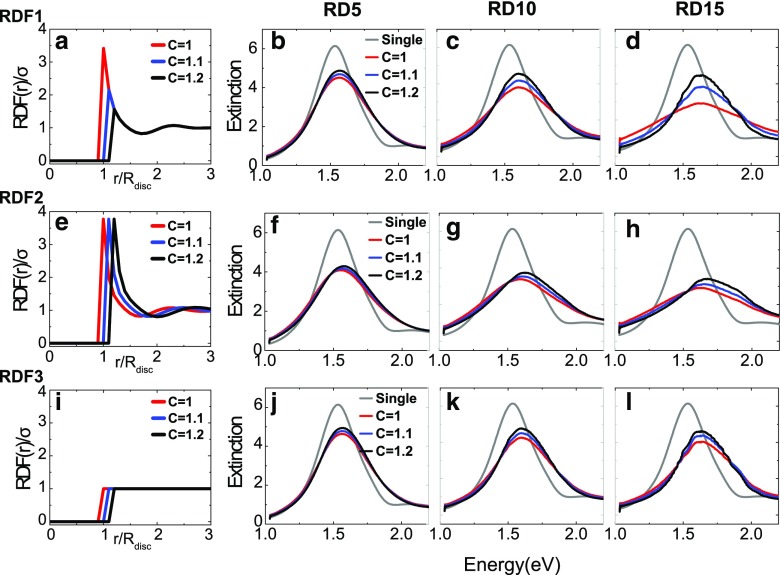
Extinction spectra at normal incidence calculated by CDA in the random cases as a function of the specific shape of the RDF as shown in the first column for a density of 15 discs/μm^2^ (*top row*, RDF1; *middle row*, RDF2; and *bottom row*, RDF3) for different values of *C* and particle densities (*columns from left to right* 5, 10, and 15 discs/μm^2^). The LSPR of a non-interacting particle (labeled “Single”) is also added for comparison. Here, “extinction” refers to the extinction efficiency *Q*_*ext*_ obtained numerically

The RDF defining the particle distribution in the meta-glasses can be changed by two parameters: *C*, the minimum nearest neighbor interaction distance (when the particles are touching, *C = 1* and the particle centers are separated by *d* = 2*R*_*disc*_), and how the RDF oscillates at small center-to-center distances before it reaches the averaged particle density above *r* = 3*R*_*disc*_ (see Fig. 3). In order to determine the influence of the shape of the RDF on the different optical properties of the structure, we calculate the extinction for three different functions, called RDF1, RDF2, and RDF3. RDF1 is a modified version of RDF_*exp*_, where RDF1(*r/R*_*disc*_) = RDF_*exp*_(*r/R*_*disc*_) for *r/R*_*disc*_ *≥ C* and otherwise RDF1(*r/R*_*disc*_) = 0; RDF2 is the shifted experimental RDF with start at different *C*; and RDF3 is the step function equal to 1 for *r*/*R*_*disc*_ ≥ *C*, indicating that a sample following this distribution will have a constant density even at the shortest distances. Figure 6a, e, i display the RDF shapes for the case *C* = 1.0, *C* = 1.1, and *C* = 1.2 only for the highest density RD15 for the sake of the readability of the graph.

**Fig. 7:**
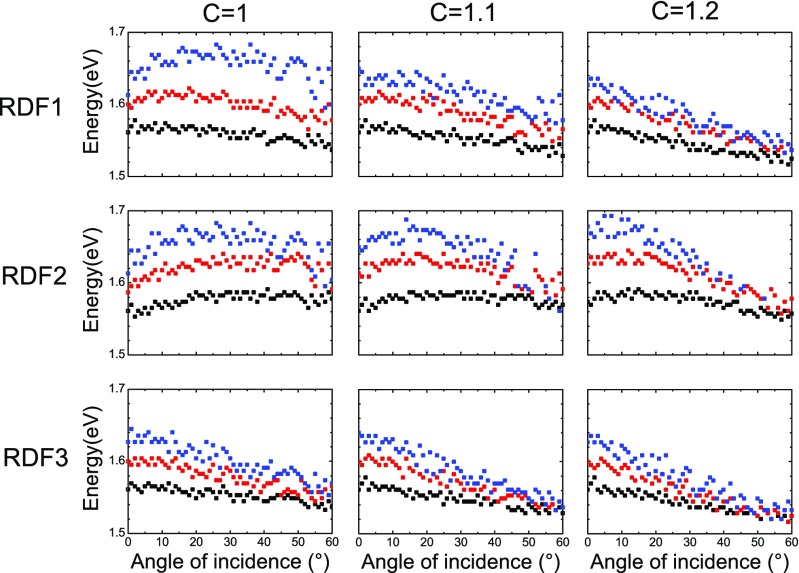
Calculated spectral position of the LSPR as a function of the angle of incidence, for different particle densities (5 discs/μm^2^ in *black*, 10 discs/μm^2^ in *red*, and 15 discs/μm^2^ in *blue*) for the different RDFs (*rows*), and as a function of C (*columns*)

In the following, we discuss the influence of the RDFs and in particular the influence of *C* on the optical response of the samples. Figure 6 illustrates the modifications of the normal incidence extinction spectra of artificial meta-glasses defined by the respective RDF. The first observation is that the position, the width, and the shape of the extinction peaks in the spectra of meta-glasses are strongly modified compared to the non-interacting case; the particle-particle interactions cannot be neglected. Apart from the particle density as main influential parameter, both *C* and the shape of the RDF influence the extinction spectra too. At low density (RD5), the shape of the RDF does not have a large influence on the shape of the extinction spectra and induce a slight intensity change. On the contrary, when the particle density increases, both *C* and the RDF have a larger influence. For RD10, the extinction increases with *C*. The resonance shift is the lowest for the step function RDF3. When the particle density reaches 15 discs/μm^2^, the value of *C* has a larger impact; a large particle density (RD15) weakens the resonance of each individual particle and induces a shift of the resonance peak. In the case of the RDF1, used for our fabricated samples, larger nearest neighbor distances induce an increase of the resonance strength towards that of the non-interacting case. These results show evidence that the resonance shape, position, and strength strongly depend on the position of the particles up to a distance *r* > *3R*_*disc*_. The influence of the nearest neighbor distance *C* can be seen further by its impact on the dispersion of the plasmonic resonance. Figure 7 displays the maximum of the extinction efficiency *Q*_*ext*_ numerically extracted from the calculated extinction coefficients as a function of the incidence angle of the light beam on the nanoparticle arrays, for all particle densities considered here, for the different RDFs and as a function of *C*. The small spread in the points represented in Fig. 7 is due to the numerical evaluation of the spectral position of the maximum of the LSPR. The calculated extinction presents some high-frequency oscillations due to the number of particles participating to the signal, which are not numerically removed and lead to a small fluctuation in the estimation of the maximum position. These numerical errors are small enough not to influence the overall trend. The simplest case is that of RDF3, the step function. Here, the dispersion is independent on the value of *C*, as expected due to the flatness of the RDF, and shows an increase of the dispersion slope with particle density, as seen experimentally in Fig. 5. When the RDF gets modified in the nearest environment of the particles, the slope of the dispersion curves changes as well. In the case of RDF2, when *C* = 1, for all the densities, the dispersion starts with a blue shift before getting flatter or turn to a red shift in the case of the highest particle density. The position of this turning point approaches normal incidence as the nearest inter-particle distance increases, until reaching the limit described by the step function. This is expected since, as *C* increases, the dispersion tends towards the case of the step function and the difference of resonance position as a function of the density is reduced. This confirms the crucial role of the minimal nearest neighbor distance on the resonance of closely packed nanostructures. The exact value of the minimum nearest neighbor influences the behavior mostly at small angles of incidence. Therefore, the measured energy minima monotonously decrease (Fig. 5), whereas in the calculation, in the case of the denser sample, it first increases then decreases due to a deviation of the experimental *C* towards larger values. Apart from this small difference, the dispersion is qualitatively well reproduced by the calculations. Now, we have identified that the particle density, the nearest neighbor distance *C*, and the specific shape of the RDF play a role in the complete optical response of the meta-glasses and we have verified that the CDA model is supported by the experiments.

## Conclusion

In conclusion, this work sheds light on the role of the specific particle distribution on the far-field optical properties of metallic particle arrays. Combining experiments performed with high quality samples and CDA calculations, we have identified the role of the different parameters such as ordered/random particle arrangement, particle density, nearest neighbor distance, local density of nearest neighbors, and global particle density at long distances on the position, shape, and dispersion of the interacting particle resonance. In all the investigated cases, the interacting particle resonance is lower in strength compared to the isolated, non-interacting case except in the case of the periodic arrays when the excitation of surface lattice resonances reshapes the extinction and increases drastically the extinction close to the RWA. Away from the RWA, the behavior of the periodic arrays is similar to that of the random case and the particle density in the near and long range plays a more important role than the exact position of the particles. Particularly, it has to be noted that random arrangements of closely packed particles deviate significantly from the non-interacting case. It is found that increased long-range particle density induces a blue shift and a broadening of the resonance as well as a decrease in the extinction per particle due to increased collective radiative coupling. On the other hand, the density of particle in the nearest neighborhood influences the position width and shape of the resonance; an increase in the near-range density decreases the overall extinction and reduces the resonance shift. As for the dispersion, while the resonance of an isolated nanoparticle is not dispersive, the dispersion increases with the number of contributing nanoparticles. The nearest neighbor distance *C* has a small influence on the shape and sign of the dispersion. However, even though the shape of the RDF, i.e., the local distribution of particles in the near range, does not influence much the position of the resonance at normal incidence, it has a strong influence on the resonance dispersion. The systematic description of the individual aspects of the particle arrangement on the extinction spectra of a nanoparticle array allows us to understand small changes in the expected extinction spectra, and can have impact on areas where a precise control of the resonance is necessary, in particular in the sensing domain.

## Experimental Section

### Fabrication

The samples were prepared using a Jeol JBX6300FS electron beam lithography system. The arrays of nanodiscs were fabricated on a fused silica substrate. Two layers of PMMA with different sensitivity were spin coated on the substrate to obtain a resist mask with undercut, which improves the lift-off of the Au film. First, PMMA 200 k 3.5 % was spin coated at 6000 rpm for 35 s and then prebaked on a hot plate for 4 min at 160 °C. In the same way, PMMA 950 k 1.5 % was spun on the sample and baked. The patterns consisting of 500 μm by 500 μm unit cell was repeatedly exposed into the resist to cover an area of 1.5 × 1.5 mm^2^; a large patterned area is crucial to perform optical measurements with a well-defined ***k*** (parallel beam), in particular at high angles of incidence. The exposure parameters were 100 kV of acceleration voltage, 1 nA of beam current, shot pitch 4 nm, and a dose of 1300 μC/cm^2^. The sample was developed in a methyl isobutyl ketone (MIBK) and isopropanol (IPA) mixture of 1:3 for 15 s at 22 °C. The development was stopped by putting the sample for 15 s in IPA and blow-dry with N_2_ gas. Thermal evaporation was then used to deposit a 1-nm-thick adhesion layer of Ti with an evaporation rate of 1 Å/second and 30 nm of Au with an evaporation rate of 2 Å/second. Finally, a 3 h-lasting N-ethyl-2-pyrrolidone (NEP) bath at 80 °C as well as followed by ultrasonic agitation applied at low power for few seconds at the end of the lift-off process. The sample was rinsed in acetone and isopropanol and then dried with N_2_. The *x* and *y* coordinates of the random lattice is formed by two randomly generated numbers; before accepting these coordinates, it is checked whether another point is located within a minimum distance of 210 nm. If yes, these coordinates are discarded. The point coordinates are generated until the desired density is reached. The large area is divided in smaller zones, thus allowing parallelization of the generation process. The accumulation of discs at the edge of the connecting border between two zones is prevented by imposing a minimum distance from the disc edge to the field border of 10 nm. Proximity error correction is performed in order to take the varying particle distance into account.

### Measurements

The transmittance measurements were performed with a variable-angle transmission setup using p-polarized incident light. The light from a broad-band Xenon source is wavelength selected by a scanning monochromator and modulated at (frequency) for subsequent lock-in detection. An optical fiber of 100 μm core diameter is used to couple the incident light beam from the monochromator to the input unit where the light is polarized. The spot size is reduced to a diameter of approximately 0.6 μm at normal incidence by a set of lenses. The light beam is collimated with a wavevector divergence smaller than 0.15°. Transmittance measurements are performed at room temperature in the spectral range between 350 nm (3.54 eV) and 1500 nm (0.826 eV) with a resolution of 1 nm, varying the angle of incidence in a θ–2θ configuration, from 0 to 60° in steps of 2°. The light transmitted through the sample is polarization selected by an analyzer selecting the p polarization only and it is collected by two photodiodes (Si and InGaAs) which cover the measured visible and near-infrared frequency range.

### Calculations

A MATLAB script was used to implement the CDA equations as described in the text. The particles were modeled as ellipsoids with in-plane radius 95 nm and out-of-plane radius of 15 nm (which corresponds to a disc thickness of 30 nm). The nanoparticles, fabricated on top of a silica substrate, are embedded in air. They are not in a homogeneous environment, as no matching liquid was used. Therefore, the nanoparticle will be influenced by both the underlying substrate and the surrounding air. In order to capture this, we have calculated the response of the particles as fully embedded in an environment of refractive index *n* = 1.25, which is chosen by taking the average value between the refractive index of air (*n*_air_ = 1.0) and substrate (*n*_subtrate_ = 1.49). However, the positions of the RWA coming from the air side or from the substrate side as respectively simulated with the appropriate value of the refractive index (*n*_air_ = 1.0 and *n*_subtrate_ = 1.49). The permittivity spectra for gold were taken from Palik [33]. For the ordered samples, periodicities *p* of 439, 312, and 262 nm were used, respectively, for the samples OD5, OD10, and OD15. Two thousand particles were taken into account for the regular calculations in the ordered case. In the random case, particles were sought for up to a distance corresponding to 500 particle diameters from the central particle. Ten slices were considered between each particle distance. For each slice, the number of present particles is determined by the value of the radial density function at the corresponding distance from the central particle. The resulting number of considered particles is similar to the total number of particles considered in the ordered case. The extinction cross section was calculated as a function of the angle of incidence between 0 and 60° with a pitch of 1°.
